# Impact of Respiratory Multiplex Polymerase Chain Reaction (PCR) on Antibiotic Stewardship: A Real-World Experience

**DOI:** 10.7759/cureus.81620

**Published:** 2025-04-02

**Authors:** Mahuya Bhattacharyya, Subhash Todi, Ananya Saha

**Affiliations:** 1 Department of Critical Care Medicine, Advanced Medical Research Institute (AMRI) Hospitals, Dhakuria, IND; 2 Department of Clinical Pharmacy, Advanced Medical Research Institute (AMRI) Hospitals, Dhakuria, IND

**Keywords:** antibiotic de-escalation, antibiotic optimization, antibiotic stewardship, hospital mortality, multiplex pcr biofire, respiratory sepsis

## Abstract

Background: This study examined whether the use of the multiplex PCR (BioFire-FilmArray [bioMérieux, Marcy-l'Étoile, France]) (BF) technique reduced or optimized antibiotic use in patients admitted to the intensive care unit (ICU) with respiratory sepsis.

Methods: This retrospective observational study included adult patients with pulmonary sepsis admitted to the ICU, where the BF test was performed using lower respiratory samples. The primary outcome measure was any appropriate antibiotic change guided by BF within 24 hours of sending samples. Hospital mortality and length of stay (LOS) were compared between the two groups: the group in which antibiotics were appropriately changed within 24 hours of sending samples for BF and the group in which they were not.

Results: A total of 117 patients with community- and hospital-acquired respiratory sepsis were included in this study. The mean APACHE IV score was 70.5±27.2, and 34 (29.1%) patients were in shock. BF was negative in 31 samples (26.5%), whereas culture was negative in 63 samples (53.8%). BF-guided de-escalation, escalation, and no change in antibiotics were indicated in 62 episodes (53%), 41 episodes (35%), and 14 episodes (11.9%), respectively. However, these changes were achieved in 15 episodes (24.2%), 40 episodes (97.6%), and 14 episodes (100%), respectively (p<0.0001). Hospital mortality and LOS were lower in cases where antibiotic alteration was indicated and performed, compared to cases where it was not (nonsignificant).

Conclusion: The identification of the causative agent using BF was higher. Achieving the appropriateness of antibiotics through escalation or continuation of the same antibiotics was more common than de-escalation. Appropriate early modification of antibiotics was associated with a decreased trend in-hospital mortality and LOS.

## Introduction

Antibiotic stewardship aims to combat antibiotic resistance by optimizing antimicrobial medication use and reducing unnecessary and inappropriate antibiotic prescriptions. The widespread use of broad-spectrum antibiotics in healthcare management has led to the emergence of several resistant bacterial strains and opportunistic infections such as *Clostridioides difficile* and fungi. Sepsis management relies predominantly on early and appropriate antibiotics. In such a scenario, it is imperative to identify the causative agent at the earliest and, preferably, its resistance pattern. Culture and antimicrobial susceptibility testing, the gold standard test to identify any microorganism, is time-consuming, with a turnover time of 48-72 hours. Studies have reported culture-negative sepsis ranging from 30% to 60% in different cohorts [1-3]. Under such circumstances, even if the culture report is available, it is of little use in decision-making regarding antibiotic choice, be it escalation and de-escalation or continuation and discontinuation. Furthermore, routine culture may not be able to isolate the organism if it is a virus, a not-easily-culturable bacterium, or if the colony count of the organisms has significantly decreased due to prior partial antibiotic treatment [4-6]. If these uncertainties regarding the causative organism are addressed, clinicians would be more confident in treating lower respiratory tract infections with fewer antibiotics.

If molecular techniques can be used to identify microorganisms reasonably rapidly, antibiotics can be changed from the second dose itself, if needed. Most of these techniques exhibit high sensitivity, specificity, and diagnostic yield. The BioFire Film Array respiratory panel [bioMérieux, Marcy-l'Étoile, France]) (BF) platform has a diagnostic yield of up to 90% compared with sputum culture with a diagnostic yield of ≤50% [7,8]. Compared with the culture of respiratory samples, BF can detect a wider range of pathogens. Multiple organisms, including viruses and bacteria, which are not so easy to grow in common culture media, can be detected in one sample. Genetic resistance patterns can be identified exactly, though not other resistance mechanisms. The benefits of molecular diagnostics for respiratory tract infections have recently been discussed in various literature. However, the utilization of molecular diagnostics and the reduction of antibiotic usage are yet to be witnessed in clinical practice. BF could potentially overdiagnose infections and identify colonizers, leading to antibiotic overuse, particularly in cases of respiratory tract infection. Hence, interpreting the test results in an appropriate clinical context, translating the findings into decisions regarding the choice of antibiotics, and a smooth implementation are challenging tasks for the hospital infection control team. This study examined whether the use of the BioFire Film Array technique helped reduce or optimize antibiotic use in patients admitted to the intensive care unit (ICU) with respiratory tract infections and whether appropriate changes in antibiotics had any effect on hospital mortality and length of stay (LOS).

## Materials and methods

Design, patients, and data collection

This retrospective observational single-center study was conducted in the mixed medical-surgical ICUs of a tertiary care multidisciplinary hospital between November 2019 and December 2021. It included patients over 18 years of age who underwent a BF test on lower respiratory samples for sepsis identification. All participants were admitted with acute-onset respiratory sepsis, diagnosed by treating physicians, necessitating microorganism identification and antibiotic therapy. Patients with incomplete data or those for whom treatment was withheld or withdrawn were excluded. Respiratory specimens comprised sputum, endotracheal aspirate, and bronchoalveolar lavage fluid. The quality of sputum samples was evaluated by analyzing the number of squamous epithelial cells and neutrophils observed per low-power field, ensuring the reliability of the samples for further diagnostic testing. A high number of epithelial cells does not necessarily indicate inflammation and may instead suggest contamination with saliva. Therefore, for analysis, sputum samples were selected based on having fewer than 10 squamous epithelial cells per low-power field (<10/lpf) and more than 10 neutrophils or pus cells per low-power field (>10/lpf) [9]. Demographic data and outcome parameters were collected from the departmental electronic database, while microbiologic and antibiotic usage data were retrieved from the hospital information system. Demographic parameters included age, sex, APACHE IV score, septic shock, and classification of sepsis as community-acquired or nosocomial. A BF test performed within 48 hours of admission was categorized as community-acquired sepsis, whereas tests conducted after 48 hours were classified as nosocomial sepsis. Organisms identified via both BF and culture were documented, along with resistance patterns determined through BF. Culture reports provided the names of identified organisms, which were used to assess concordance with the BF findings.

Outcome and primary exposure

Concordance between the BF and culture results for respiratory samples was assessed and categorized into four groups: both BF and culture positive, both negative, BF positive and culture negative, and culture positive and BF negative. Antibiotic modification within 24 hours of sending samples for BF was recorded. Bedside antibiotic decisions were made by ICU consultants, with input from microbiologists or treating physicians. The initial antibiotic selection was guided by the hospital antibiogram and aligned with standard treatment guidelines. From the available data, the study investigators assessed whether a change in ongoing antibiotics was indicated after the BF report. Changes in antibiotics included escalation, de-escalation, or no change. A change in antibiotics was considered when indicated after comparing the organism identified via BF, its resistance pattern, and the ongoing antibiotics. If the organism belonged to the gram-negative group, discontinuation of antibiotics covering gram-positive organisms was considered appropriate, and vice versa. The presence or absence of one or more resistance genes guided changes in antibiotic therapy. When a more resistant gene is identified, antibiotic therapy should be escalated to include a higher-tier antibiotic specifically effective against the resistant organism. Conversely, in the absence of resistance genes, therapy should be adjusted to a narrower-spectrum or lower-tier antibiotic, aligning treatment with the identified pathogen's susceptibility. In cases where only a virus was identified, discontinuation of all antibiotics and the addition of an antiviral agent, if available, was deemed appropriate. The process for selecting the optimum or preferred antibiotic is outlined in Table 1. Any other available culture report at that point was also considered in determining appropriate antibiotics. Blood and urine were the other samples considered, as antibiotics were sometimes continued for sepsis of non-respiratory origin. However, further modifications to the antimicrobial agent based on subsequent respiratory culture results for the same sepsis episode were not addressed in this study. The study investigators deemed a change in antibiotics appropriate if it was implemented as indicated based on the BF report. The primary outcome measure was the modification of antibiotics within 24 hours of sending the sample for the BF test. Patients were categorized into two groups based on antibiotic changes: 1) the appropriate group, where an indicated change was performed, and 2) the inappropriate group, where an indicated change was not implemented. Secondary outcome measures included hospital mortality and length of stay (LOS), which were compared between these two groups. Multivariate logistic regression analysis was conducted to identify variables significantly associated with mortality.

**Table 1:** Antibiotic decision based on BF result CTX-M: a group of extended-spectrum β-lactamases, NDM: New Delhi metallo-beta-lactamase, OXA: oxacillinase, KPC: *Klebsiella pneumoniae carbapenemase* *Antibiotic selection was guided by the specific organism identified along with its resistance pattern, local antibiogram for respiratory sepsis, and standard guideline for antibiotic use in respiratory sepsis **Pseudomonas aeruginosa: Dual antibiotic therapy, if utilized, was considered appropriate. **Acinetobacter baumannii: Dual antibiotic therapy, including sulbactam, was considered appropriate.

Resistance gene identified by BF	Optimum/preferred antibiotic*
CTX-M	Carbapenems
NDM, IMP, OXA including OXA-48**	Ceftazidime – Avibactam ± Aztreonam
	Tigecycline
	Minocycline
	Polymyxin B
	Colistin
KPC**	Polymyxin B
	Colistin
	Ceftazidime – Avibactam
mec-A/C positive	Vancomycin
	Teicoplanin
	Linezolid
	Daptomycin
	Ceftaroline
mec- A/C negative	Oxacillin/Flucloxacillin
	Cefazolin
	Cephalexin

Statistical analysis

The categorical variables were expressed as the frequency of events or the number of patients and their percentage and compared across the groups using Pearson’s chi-square test for independence of attributes or Fisher’s exact test as appropriate. The continuous variables were expressed as mean, median, and standard deviation and compared across the groups using the Mann-Whitney U test, as the data did not follow a normal distribution. Multivariate analysis was performed using binary logistic regression. The statistical software IBM Corp. Released 2017. IBM SPSS Statistics for Windows, Version 25.0. Armonk, NY: IBM Corp. was used for the analysis. An alpha level of 5% was considered, i.e., if any p-value was <0.05, it was considered significant.

Ethical consideration

This study was approved by the Institutional Ethics Committee. All data analyzed in this study were de-identified and anonymized. The requirement for informed patient consent was waived.

## Results

The study screened 140 patients, of which 23 were excluded because of withdrawal of treatment, inadequate quality of sputum samples, and missing or duplicate data, and finally, 117 patients were included in the analysis. All patients had respiratory system involvement, with a diagnosis of COPD/asthma exacerbation, pneumonia, or acute lung injury on inclusion. These patients were sick enough to be admitted or transferred to ICUs. The demographic characteristics of the study population are listed in Table 2. The study population included patients who were either suffering from or had suffered from SARS-CoV-2 infection (47.8%, n=56). Those identified as having secondary pneumonia in this study either were admitted after a COVID-19 infection or developed another pulmonary sepsis during convalescence from COVID-19 during the same hospital stay. The study included both community-acquired and nosocomial sepsis.

**Table 2:** Demographic characteristics of the study population *APACHE IV score: Acute physiology and chronic health evaluation IV score

Variables	Total patients (n=117)
Age, years (Mean ± SD)	65.4 ± 15.8
Male sex n(%)	70 (59.8)
APACHE IV score * (Mean ± SD)	70.5 ± 27.2
Septic shock n(%)	34 (29.1%)
Nosocomial sepsis n(%)	41 (35%)
Diagnosis on inclusion	
COVID 19 ± secondary pneumonia n(%)	56 (47.8%)
Pneumonia n(%)	53 (45.2%)
Others n(%)	8 (6.8%)

Pathogen detection

The lower respiratory samples for BF and culture included 44 and 42 sputum samples (37.6% and 35.9%, respectively), 71 and 66 endotracheal tube aspiration samples (60.7% and 56.4%, respectively), and 2 and 3 bronchoalveolar lavage (BAL) samples (1.7% and 2.6%, respectively). BF was negative in 31 samples (26.5%). The culture report was unavailable in seven cases (5.9%) and negative in 63 cases (53.8%). Concordance (both BioFire and culture were positive or negative) was observed in 61 patients (52.1%), and the results are elaborated in Table 3. Blood and urine cultures were positive in 12.8% (n=15) and 9.4% (n=11) of the cases, respectively.

**Table 3:** Pathogen detection in respiratory samples CTX-M: A group of extended-spectrum β-lactamases, NDM: New Delhi metallo-beta-lactamase, OXA: Oxacillinase, KPC: *Klebsiella pneumoniae carbapenemase*, IMP: Imipenem-resistant pseudomonas, VIM: Verona integron-encoded metallo-beta-lactamase, mecA/C: Genes responsible for methicillin resistance in *staphylococci* *Detected by BioFire ** *Enterobacteriaceae* included *Escherichia coli*, *Klebsiella pneumoniae*, *Klebsiella oxytoca*, and *Enterobacter cloacae* # Percentage calculations were not performed, as multiple organisms were often identified from a single respiratory sample

Variables	Total patients (n=117)		
Lower respiratory samples	BioFire n (%)	Culture n (%)	
Sputum	44 (37.6)	42 (35.9)	
Endotracheal tube aspiration	71 (60.7)	66 (56.4)	
Bronchoalveolar lavage	2 (1.7)	3 (2.6)	
Concordance between BF and culture results n (%)	61 (52.1)		
BF positive, culture positive(concordant) n (%)	41 (35)		
BF negative, culture negative(concordant) n (%)	27 (23.1)		
BF positive, culture negative(discordant) n (%)	45 (38.5)		
BF negative, culture positive(discordant) n (%)	4 (3.4)		
Single organism n (%) *	30 (26.5)		
More than one organism n (%) *	53 (45.3)		
Organisms detected by BF	n=episodes of identification #		
Enterobacteriaceae**	46		
Pseudomonas aeruginosa	28		
Acinetobacter calcoaceticus baumannii complex	29		
Haemophilus influenzae	6		
Staphylococcus aureus (methicillin sensitive))	9		
Staphylococcus aureus (methicillin resistant)	2		
Streptococcus pneumoniae	9		
Streptococcus agalactiae	3		
Serratia marcescens	2		
Moraxella catarrhalis	1		
Proteus mirabilis	1		
Virus			
Influenza A virus	17		
Respiratory syncytial virus	4		
Human Rhino/ Enterovirus	4		
Coronavirus (other than SARS-CoV-2)	3		
Parainfluenza virus	1		
Adenovirus	1		
Resistance gene identified by BF *	n=all samples #		n=>48 hours sample #
CTX-M	35		13
IMP	11		3
KPC	6		3
NDM	43		22
OXA-48	24		11
VIM	22		7
mecA/C	11		0
Organisms identified by culture	Episodes n (%)		
Culture not available	7 (5.9)		
Culture negative	63 (53.8)		
Acinetobacter baumannii	11 (9.4)		
Klebsiella pneumoniae	13 (11.1)		
Pseudomonas aeruginosa	6 (5.1)		
Aspergillus fumigatus	7 (5.9)		
Escherichia coli	3 (2.5)		
Burkholderia cepacia	2 (1.7)		
Stenotrophomonas maltophilia	3 (2.5)		
Serratia marcescens	2 (1.7)		

Antibiotic modification and outcome

A change in antibiotics within 24 hours of sending the sample for BF was observed in 69 patients (59.0%). De-escalation was indicated in 62 episodes of sepsis (53%); however, it was implemented only in 15 episodes (24.2%). Escalation was indicated in 41 episodes of sepsis (35%) and was implemented in 40 episodes (97.6%). No change in antibiotics was deemed appropriate in 14 episodes of sepsis (11.9%) and was achieved in 14 episodes (100%). Details of the change in antibiotics are furnished in Table 4. Blood culture contributed to de-escalation in three cases (p=0.06), with the cultures identifying *Acinetobacter lwoffii* (n=1) and *Staphylococcus hominis* (n=2). Urine culture results did not facilitate antibiotic de-escalation. Neither blood nor urine cultures had any influence on escalation, nor did they prompt any new changes in antibiotic therapy.

**Table 4:** Antibiotic modification * A p-value of < 0.05 was considered significant § Detected by BF test ∞ Blood culture not available and negative, n=30, 72, respectively ∞∞Urine culture not available and negative, n=34, 72, respectively

Variables	Total patients n (%)	p-value	Escalation of antibiotics achieved n (%)	De-escalation of antibiotics achieved n (%)	No change in antibiotics achieved n (%)	p-value	Appropriate antibiotic change n (%)	p-value
Antibiotic modification within 24 h of sending BF	69 (59.0)	0.001*	40 (97.6)	15 (24.2)	14 (100)	0.0001*	69 (59.0)	0.01*
Single organism^§^	30 (25.6)	-	10 (33.3)	3 (10)	17 (56.6)	-	15 (50)	-
More than one organism^§^	53 (45.3)	-	33 (62.2)	9 (17)	11 (20.7)	-	4 (7.5)	-
No organism^§^	34 (29.0)	-	6 (17.6)	3 (8.2)	25 (73.5)	-	5 (14.7)	-
Septic shock	34 (29.1)	-	18 (52.9)	5 (14.3)	11 (32.3)	-	21 (61.7)	-
Nosocomial sepsis	41 (35)	-	19 (44.1)	2 (4.6)	20 (48.7)	0.01*	21 (48.8)	-
BF positive and culture negative	45 (38.4)	-	16 (35.5)	10 (22.2)	19 (42.2)	-	30 (66.6)	-
Blood culture positive∞	15	-	-	3	-	-	-	-
Urine culture positive∞∞	11	-	-	0	-	-	-	-

Crude hospital mortality was 53.8% (n=63) in the study population and was the highest in the subgroup in which the antibiotic was escalated (63.4%, n=26), followed by the no de-escalation and no change groups (53.2%, n=33, and 28.6%, n=4, respectively) (p-value 0.07). Hospital mortality and LOS were also higher in the inappropriate antibiotic group, although it was not statistically significant (Table 5). Appropriate changes in antibiotics did not have a significant impact on crude hospital mortality in the multivariate analysis (p > 0.5, OR 0.77, CI 0.3-1.9) compared with the APACHE IV score and the presence of a shock state (p< 0.003, OR 1.03, CI 1.01-1.05 and p< 0.03, OR 3.03, CI 1.06-8.6, respectively).

**Table 5:** Outcome of the study population A p-value of < 0.05 was considered significant

Variables	Total patients n (%)	Hospital mortality n (%)	p-value	Hospital LOS days, (mean ± SD)	p-value
Appropriate group	69 (59)	36 (52.2)	-	13.3 ± 8.6	-
Inappropriate group	48 (41)	27 (56.3)	-	17.9 ± 14.2	-
Chi-square test	-	-	0.66	-	0.14

## Discussion

Pulmonary sepsis is associated with significant morbidity and mortality, especially if not treated with timely and appropriate antibiotics, as concluded by several studies in the last decade [10-12]. This condition exhibits a complex pathogenic and microbiological spectrum, with causative organisms mostly falling in the category of viruses, bacteria, at times a combination of both, or a few specific fungi, with each situation warrants a different treatment. The sooner the organism and its resistance pattern are identified, the earlier the appropriate antimicrobial agent can be used for the treatment [13,14].

Our study analyzed 117 patients with pulmonary sepsis. The mean APACHE IV score was 70.5 ± 27.2 (SD), and approximately one-third of the patients were in shock, indicating that the cohort included many critically ill ICU patients. The identification of organisms was significantly higher via BF than culture, with culture-negative events accounting for 53.8% (n=63) and BF-negative events accounting for 26.5% (n=31) of all recorded cases. Varied findings have been obtained in studies addressing the yield of BF in hospital-admitted patients [15,16]. Qian et al. found that FA-RP (Film Array Respiratory Panel) could detect pathogens in 76.8% of all specimens in hospital-admitted patients, whereas few other studies found the detection rate to be much lower in community-acquired pneumonia (38.6%) [7,8].

Compared with BF, identifying multiple pathogens using the culture method is relatively rare, as conventional sputum culture often fails to detect co-infections and atypical pathogens. However, infections involving multiple pathogens, such as a virus and a bacterium or two bacteria, are not uncommon. In our study cohort, BF detected mixed infections with two or more pathogens in 45.3% (n=53) of cases, a finding consistent with reports from other studies [5,17]. In such cases, if the bacteria identified are resistant to the antibiotics being administered, the patient's clinical condition may deteriorate after an initial period of improvement, potentially complicating their recovery. Concordance between FA-PNEU (Film Array Pneumonia) and culture was 90.1% in the study conducted by Molina et al. [18], but it was rather low in our study (n=61, 52.1%). Variations in the concordance rate may be attributed to the types of organisms identified in each specific cohort. If viruses or non-cultivable organisms are present in a patient cohort, the level of discordance between diagnostic tools is likely to increase. Our study cohort reported the identification of viruses in 25.6% (n=30) of cases. BF-positive and culture-negative discordance may play a significant role in guiding antibiotic usage. In our patient population, this type of discordance had the highest occurrence, accounting for 38.5% (n=45) of the cases. 

This study focused on identifying modifications in antibiotic therapy within 24 hours of sending samples for the BF test as the primary outcome and assessing the appropriateness of these changes. Antibiotic modification occurred in 69 cases (59.0%) (p 0.001), with appropriate antibiotics, as determined by the BF test, being used in the same proportion of cases (n=69, 59.0%) (p 0.01). Further analysis revealed that the incidence of appropriate de-escalation was relatively low (n=15, 24.2%) compared with escalation or no change (n=40, 97.6% and n=14, n=14,100%, respectively) (p<0.0001). Achieving the appropriateness of antibiotics through escalation or continuation of the same antibiotics was more common than de-escalation. The cases where de-escalation was considered appropriate according to the BF test but was not done could be attributed to several reasons, such as the shock state, worsening clinical condition owing to cytokine storm and other immunological damage, presence of more than one organism or resistance mechanism, other ongoing infections requiring different antibiotics, antibiotic decision not entirely depending on the BF result, or the patient improving with existing antibiotics. De-escalation was even lower in nosocomial infection (n=2, 4.6%). Rapid identification of pathogens after the introduction of the FilmArray has been viewed by several researchers as a method to decrease the percentage of patients receiving antibiotics and also their duration. However, several studies have also failed to prove this hypothesis [7,8,19,20].

The decision for appropriate antibiotics based on BF was challenging in cases where either multiple organisms were identified or no organism was identified. When more than one organism was detected, antibiotics were chosen to target all or most of them. Conversely, when no organism was identified via BF, antibiotics were not altered. There is currently a lack of definite guidelines or consensus on strategies to reduce the number of antibiotics in such scenarios. In our study cohort, appropriate modification of antibiotics following the BF test was achieved in only 7.5% of cases (n=4) with multiple organisms identified and 14.7% of cases (n=5) with no organisms identified. A single organism was identified in 25.6% of patients (n=30), and an appropriate change in antibiotics was implemented in 50% of these cases (n=15). Although this rate remains low, it was better than the other two scenarios. More than half of the patients in the septic shock subgroup (n=21, 61.7%) and those with nosocomial infection (n=21, 48.8%) had appropriate changes in antibiotics. The highest de-escalation happened in BF-positive and culture-negative groups, achieving 66.6% (n=30) of appropriate antibiotic use. However, none of these subgroup analyses reached statistical significance. Results vary across studies depending on the variables measured and subgroups analyzed. For instance, Qian et al. observed that positive FA‐RP results significantly reduced both antimicrobial and antibiotic/antifungal-defined daily doses (DDD) compared with cases where the result was negative. These researchers opined that the study findings strongly indicated that the results of the FA‐RP test could aid decision-making regarding antibiotic usage [7]. Brendish et al. concluded that routine molecular point-of-care testing for viruses in adults with acute respiratory illness increased the proportion of patients receiving single doses or brief courses of antibiotics compared with the control group [19]. In a study by Pickens et al., antibiotic de-escalation was implemented in 65.9% of the patients based on the results of the LRT Panel, of whom 69% had unnecessary methicillin-resistant *Staphylococcus aureus* coverage and 64% had *Pseudomonas aeruginosa* coverage [21]. In such complex clinical situations, interpreting BF results is critical, and appropriate implementation in different patient categories is expected to reduce antibiotic use [22].

Crude hospital mortality was 53.8% (n=63), with the highest rate observed in the subgroup where antibiotics were escalated (63.4%, n=26). This was followed by the no-de-escalation group (53.2%, n=33) and the no-change group (28.6%, n=4). Hospital mortality and LOS were higher in groups where antibiotic alteration was indicated but not implemented. This finding aligns with observations reported in other studies [4,23]. Outcome parameters showed only a trend and were not statistically significant, which could be ascribed to several factors, including the small number of patients in our study cohort and the varied attributable causes of death across the study population. In the multivariate analysis, appropriate changes in antibiotics did not have a significant impact on crude mortality (p=0.5), in contrast to the APACHE IV score and the presence of a shock state, which were significant (p=0.003 and 0.03, respectively).

Our study has several limitations. First, the data were collected during a period of high community prevalence of SARS-CoV-2 infection, which limits the generalizability of the results to patients not recently exposed to the virus. Second, the BF panel for lower respiratory samples did not include SARS-CoV-2, potentially leading to missed infections. Third, the study population size was insufficient to significantly impact outcomes, although it showed a trend toward improvement. A clearer understanding might be achieved with a larger cohort of patients. Fourth, the appropriateness of antibiotics was primarily based on the BF report for the study, without considering the patient's clinical condition. Fifth, the study did not address whether antibiotics were further modified following the availability of culture results. The main strength of this study is its inclusion of a diverse subset of critically ill patients, encompassing those with community-acquired and nosocomial sepsis-a group that has not been well addressed in previous BF studies.

## Conclusions

The analysis of 117 patients with respiratory sepsis revealed several key insights. The identification of organisms was significantly higher via BF compared to culture. Appropriate antibiotics, as determined by BF, were used in 59% of the cases. However, the incidence of appropriate de-escalation was significantly lower than that of escalation or no change (p=0.0001). Hospital mortality and LOS were higher in groups where antibiotic alteration was inappropriate, though this finding was not statistically significant. This study emphasized the impact of BF on rapid decision-making regarding antibiotic optimization. When applied judiciously, the BF respiratory panel has the potential to serve as a powerful decision-making tool for patient management. It could improve the detection of clinically relevant pathogens, enhance antibiotic utilization, and influence patient outcomes. Future studies should aim to explore how BF can further optimize antibiotic use in critically ill patients with multiple pathogens in respiratory samples.
